# MicroRNA-Like Small RNAs Prediction in the Development of *Antrodia cinnamomea*

**DOI:** 10.1371/journal.pone.0123245

**Published:** 2015-04-10

**Authors:** Yan-Liang Lin, Li-Ting Ma, Yi-Ru Lee, Shih-Shun Lin, Sheng-Yang Wang, Tun-Tschu Chang, Jei-Fu Shaw, Wen-Hsiung Li, Fang-Hua Chu

**Affiliations:** 1 School of Forestry and Resource Conservation, National Taiwan University, Taipei, Taiwan; 2 Institute of Biotechnology, National Taiwan University, Taipei, Taiwan; 3 Department of Forestry, National Chung-Hsing University, Taichun, Taiwan; 4 Agricultural, Biotechnology Research Center, Academia Sinica, Taipei, Taiwan; 5 Agricultural Biotechnology Center, National Chung-Hsing University, Taichung, Taiwan; 6 Division on Forest Protection, Taiwan Forestry Research Institute, Taipei, Taiwan; 7 Department of Biological Science & Technology, I-Shou University, Kaohsiung, Taiwan; 8 Biodiversity Research Center, Academia Sinica, Taipei, Taiwan; 9 Department of Ecology and Evolution, University of Chicago, Chicago, Illinois, United States of America; 10 Experimental Forest, National Taiwan University, Nan-Tou, Taiwan; Kunming University of Science and Technology, CHINA

## Abstract

*Antrodia cinnamomea*, a precious, host-specific brown-rot fungus that has been used as a folk medicine in Taiwan for centuries is known to have diverse bioactive compounds with potent pharmaceutical activity. In this study, different fermentation states of *A*. *cinnamomea* (wild-type fruiting bodies and liquid cultured mycelium) were sequenced using the next-generation sequencing (NGS) technique. A 45.58 Mb genome encoding 6,522 predicted genes was obtained. High quality reads were assembled into a total of 13,109 unigenes. Using a previously constructed pipeline to search for microRNAs (miRNAs), we then identified 4 predicted conserved miRNA and 63 novel predicted miRNA-like small RNA (milRNA) candidates. Target prediction revealed several interesting proteins involved in tri-terpenoid synthesis, mating type recognition, chemical or physical sensory protein and transporters predicted to be regulated by the miRNAs and milRNAs.

## Introduction

MicroRNAs (miRNAs) are a group of small non-coding RNAs commonly 21–22 nucleotides (nt) in length that have important roles in the post-transcriptional regulation of gene expression in plants and animals. They regulate a wide range of cellular processes including multicellular development, differentiation, apoptosis and stress response [1,2]. Mature miRNA regulates target gene expression negatively through complementary binding to coding sequences or the untranslated regions. In animals, imprecise pairing of miRNA and the specific target genes leads to translational inhibition, and in plants the near perfect complementarity between miRNAs and their targets leads to messenger RNA cleavage [3,4]. RNA interference (RNAi)-related gene silencing in fungi was first described in *Neurospora crassa* in 1992 [5]. RNAi pathways use three major types of small noncoding RNAs, small interference RNAs (siRNAs), microRNAs (miRNAs) and PIWI-interacting RNAs (piRNAs) to regulate diverse cellular processes. RNAi components and functions have been identified in many fungal species but no miRNA was discovered in fungi [6]. Until 2010, miRNA-like small RNAs (milRNAs) have been identified in the model fungus *Neurospora crassa*. Different to the animals and plants, *N*. *crassa* consist at least four different types of milRNA generation mechanisms with different combination of Dicers, QDE-2, QIP and an RNase III domain containing protein [7]. Accelerating with the rapid applied of next-generation sequencing (NGS) techniques, more fungi milRNAs have been published, such as: *Cryptococcus neoformans* [8], *Fusarium oxysporum* [9], *Metarhizium anisopliae* [10], *Sclerotinia sclerotiorum* [11], *Trichoderma reesei* [12] and *Penicillium marneffei* [13]. Although the milRNAs have been identified in several fungi species, the exactly functions or regulatory roles still not well understood. With more published fungi milRNAs and related researches may aid in explaining the evolution and function in eukaryotes.

*Antrodia cinnamomea* is a precious, brown-rot fungus also called “chang-chih” or “niu-chang-ku” that is only found in the wild in the inner cavity of *Cinnamomum kanehirae* Hayata. *A*. *cinnamomea* has been used as a folk medicine to cure alcohol poisoning, diarrhea, drug intoxification and liver cancer for centuries [14]. Several *in vivo* and *in vitro* studies have revealed that *A*. *cinnamomea* has diverse biological activities, such as anti-cancer, anti-tumor, anti-inflammatory, anti-hepatitis, anti-oxidative, hepatoprotective and vasorelaxation [15,16]. These reports suggest potential pharmaceutical applications for *A*. *cinnamomea*. However, *A*. *cinnamomea* can only grow on *C*. *kanehirai* Hayata, an endangered species native to Taiwan [17] and it grows at an extremely slow rate. Although laboratory culture systems can easily produce massive amounts of *A*. *cinnamomea* mycelium, certain specific, medical compounds and metabolites are reported to be deficient in the artificial cultured mycelium. In addition, fruiting body formation is difficult to induce [16,18,19].

In recent years, NGS techniques have improved the efficiency of the discovery of novel genes and understanding of differential gene expression patterns accompanying different physiological properties [20]. Although NGS techniques have been used to obtain massive amounts of genetic information including genome sequences and transcriptome analysis (RNA-seq) of many important fungi species such as, *Agaricus bisporus* [21], *Ganoderma lucidum* [22,23], *Laccaria bicolor* [24,25], *Phanerochaete chrysosporium* [26,27], data on higher basidiomycetes such as *A*. *cinnamomea*, which have an irregular plate shape and possess numerous bioactive compounds, is rare. The potential roles of milRNAs in higher basidiomycetes remain unknown. In this study, the assembly of the genome sequence, transcriptome analysis and microRNA prediction provides information about the artificial culture system, fungal physiology and secondary metabolite biosynthesis of *A*. *cinnamomea*, which may be useful for future commercial applications

## Materials and Methods

### Fungal strain and culture condition

*Antrodia cinnamomea* wild-type fruiting bodies and artificial cultured mycelium WSY-01 were provided by Prof. Sheng-Yang Wang (National Chung-Hsing University). The haploid type mycelium isolate S28 was provided by Dr. Tun-Tschu Chang (Taiwan Forestry Research Institute). The mycelium was maintained in MEA medium (2% malt-extract, 2% glucose and 2% phyto-agar if cultured in the solid state) in darkness at 24°C without shaking.

### RNA and genomic DNA preparation

The total RNA and genomic DNA preparation was as described by Lin et al. [18] and Chu et al. [28]. The small RNA preparation was extracted with PureLink miRNA isolation kit (Thermo, Waltham, MA) according to the manufacturer’s protocol.

### Next-generation sequencing and sequence assembly

Small and total RNA extracted from wild-type fruiting bodies and artificial cultured mycelium WSY-01 were used for the small RNA library and transcriptome library (RNA-seq) construction. The genomic DNA extracted from haploid type mycelium S28 was used for the genomic DNA library construction. The prepared nucleic acid (total RNA and genomic DNA) was fragmented randomly, adapter-ligated and finally sequenced using the Illumina HiSeq 2000 high-throughput platform according to the manufacturer’s protocol (Illumina, San Diego, CA). The small RNA library construction was sequenced by SOLiD next-generation sequencing platform 5500XL according to manufacturer’s protocol (Thermo, Waltham, MA).

The genomic DNA, small RNA and RNA-seq sequencing raw reads were filtered to obtain high quality clean data without adapter or duplicated sequences before assembly. Genome assembly was performed de novo with SOAPdenovo version 1.05 with parameter K set as 35 [29]. After sequence assembly, ab initio gene prediction was performed with Augustus (version 2.6.1) based on a generalized Hidden-Markov Model [30]. The small RNA high quality reads were extracted and counted by CLC Genomic Workbench (version 5.5.2; CLC bio, Denmark), RNA-seq de novo assembly was performed using the Trinity platform [31] and CLC Genomic Workbench. The contigs were further assembled by CAP3 [32] to generate unigenes.

The sequenced raw reads data in this paper have been deposited to Sequence Read Archive (SRA) database in National Center for Biotechnology Information (NCBI) under BioProject accession no. PRJNA268267, transcriptome accession no. SRR1662167 (FB) and SRR1662168 (MY), sRNA accession no. SRR1662191 (FB) and SRR1662192 (MY), genomic DNA accession no. SRR1663426.

### Transcriptome annotation and differentially expressed gene analysis

Functional annotation of the assembled unigenes was performed using the BLAST algorithm [33] against to the NCBI non redundant (nr) protein database with an e-value threshold of 1e-5. The gene ontology annotation process was performed with Blast2GO [34] and GO term classification was performed with CateGOrizer [35]. The KEGG enzymatic pathway analysis was performed with KAAS [36].

Differentially expressed gene (DEG) analysis was based on RPKM (reads per kilo bases per million reads) mapping to different RNA-seq libraries and calculated with DEGseq [37]. FDR <5 and fold change ≥2 or ≤0.5 was defined as differential expression.

### miRNA prediction and target gene prediction

The small RNA (sRNA) trimming and preprocessing was performed with CLC Genomic Workbench to remove adaptor sequences and low quality reads to leave high quality clean reads. Then the clean reads were further analyzed with CLC Genomic Workbench to extract and group the sRNA reads. These grouped sRNAs were first annotated against the miRBase database version 18 to find the conserved miRNA candidates. Next, the same grouped sRNAs were used for milRNAs prediction using the pipeline constructed by Chang in 2012 [38]. Briefly RNA folding for the contigs from RNA-seq was predicted for minimum free energy using RNAfold in the ViennaRNA package [39]. Then the high quality small RNA clean reads were remapped back onto the folded contigs. Two small RNA reads mapped to the same folded contig were kept as milRNA precursor candidates. Then the milRNA and precursor candidates were checked according to the following criteria: (1) two mapped reads appearing on the same stem structure with fewer than 4 missmatched nucleotides; and (2) read counts of milRNA larger than milRNA*.

The milRNA target prediction was performed with psRNATarget [40] in user-submitted form and the maximum expectation set as 4.0. The milRNAs and assembled unigenes were submitted to the server for target gene prediction.

### Northern blot of sRNAs

The total RNAs purified from wild-type fruiting body and mycelium were separated with 15% acrylamide/8 M urea gel and transfer to the Hybond-N+ nitrocellulose membrane (GE healthcare, Piscataway, NJ). The specific isotope probes were label with γ-[^32^P] ATP (3000 Ci/mmol, PerkinElmer, Waltham, MA) using T4 polynucleotide kinase (NEB, Ipswich, MA). Then the membrane exposed to the Amersham hyperfilm MP autoradiography film (GE healthcare, Piscataway, NJ).

### *DCL*, *QDE* gene isolation and RT-PCR

*DCL-1*, *DCL-2* and *QDE-2* (argonaute) were the three major components of the miRNA generation [6]. These gene homologs from other fungal species were collected from the NCBI database, and the BLAST algorithm was used to search against the RNA-seq constructed from *A*. *cinnamomea*. The most similar contig was used for the molecular cloning by specific primer pair. The reverse transcriptase polymerase chain reaction (RT-PCR) used RNA purified from different fermentation stages of *A*. *cinnamomea*. RT-PCR thermal cycling reaction was performed for 22–26 cycles with each cycle comprising of denaturation at 94°C for 30 s, annealing at 55°C for 30 s, and extension at 72°C for 30 s. *DCL-1*, *DCL-2*, *QDE-2* and 18s rRNA gene were monitored.

## Results

### Next generation sequencing and de novo assembly

To establish basic genetic information about *A*. *cinnamomea* the haploid type *A*. *cinnamomea* isolate S28 genomic DNA was purified, fragmented randomly and sequenced using the Illumina HiSeq 2000 high-throughput platform. The insert size of the genomic DNA fragment was 500 bp and the read length was 90 bp paired-end. The sequencing generated 1,485 M raw reads. After adapter and duplicated sequences, and low quality reads had been removed, a total of 1,350 M clean reads were used for the genome assembly. Assembly with SOAPdenovo generated 1,242 scaffolds with N50 = 84,738 bp. Maximum scaffold length was 376,440 bp, and total length was 27,717,145 bp with GC content 50.62% and 6,522 possible genes predicted by Augustus. The k-mer frequency distribution predicted the genome size to be 45.58 Mbp (S1 Fig).

To compare the gene expression of the liquid culture mycelium (MY) and the wild-type fruiting bodies (FB), RNAs from both sources were purified. Two libraries (MY and FB) were constructed separately and sequenced with Illumina HiSeq 2000. The general features of the different RNA-seq libraries are listed in Table 1. This sequencing yielded 21,670,612 and 27,691,800 raw reads from MY and FB, respectively. After removing low quality reads, 19,875,544 and 25,296,502 high quality clean reads from MY and FB, respectively, were used for contig assembly separately and combined together. RNA-seq assembly using the reads mixed from MY and FB can increased the contigs number from 14,075 to 14,490 with an average length of 1,278 bp to 1,615 bp. Finally 13,109 unigenes with an average length 1,615 bp (N50 = 2,770 bp) were obtained. The total length of the unigenes was 21,174,312 bp.

**Table 1:** General features of RNA-seq library from different states of A. cinnamomea.

	MY	FB	MY+FB
Number of raw reads	21,670,612	27,691,800	49,362,412
Number of clean reads	19,875,544	25,296,502	45,172,046
Number of contigs	14,364	14,075	14,490
Number of unigenes	-	-	13,109
Average length (bp)	1,278	1,402	1,615
N50 (bp)	2,166	2,460	2,770
Total length (bp)	18,358,638	19,737,996	21,174,312

### Functional annotation, gene ontology and differentially expressed genes

*A*. *cinnamomea* S28 genomic DNA library scaffold and RNA-seq unigene functional annotation were performed with BLAST algorithm against the nr protein database. Of 13,109 unigenes in the RNA-seq library, 7,851 unigenes (59.9%) showed sequence homology to known proteins from the nr database and 4,077 unigenes (31.1%) were matched to homologs from the KOG database. The unigenes were distributed across 239 different KEGG pathways.

**Fig 1:**
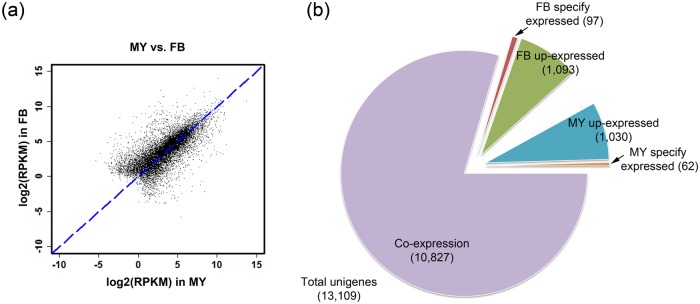
Differentially expressed gene analysis of wild-type fruiting bodies and liquid cultured mycelium of *A*. *cinnamomea*. F: wild-type fruiting bodies; M: liquid cultured mycelium. (a) Scatter plot of unigenes from *A*. *cinnamomea* RNA-seq. (b) Pie chart of the DEG distribution in wild-type fruiting bodies and liquid cultured mycelium. FDR <0.05 and fold change ≥2 or ≤0.5 were defined as differential expression.

To analyze the differential gene expression in *A*. *cinnamomea* liquid cultured MY and wild-type FB, expression abundance was calculated by the reads from different RNA-seq libraries mapped to the assembled unigenes to generate the RPKM. The relative expression value of each unigene was distributed on a scatter plot (Fig 1a). Most contigs remained near or on the dotted line and showed equal levels of expression in MY and FB. Relative expression abundance was calculated with DEGseq. Differential expression was defined as FDR <5 and fold change ≥2 or ≤0.5. Of 13,109 unigenes, 2,282 were found to be differentially expressed (Fig 1b). Of the 2,282 DEGs, 1,030 were upregulated in MY and 1,093 were upregulated in FB, and 62 and 97 unigenes were specifically expressed in mycelium or fruiting bodies, respectively (Fig 1b). The details of the unigenes expressed specifically in mycelium or fruiting bodies that have known functions are listed in S1 Table. Several proteins were found to be involved in mycelium mating, tri-terpenoid regulation and transporters.

**Fig 2:**
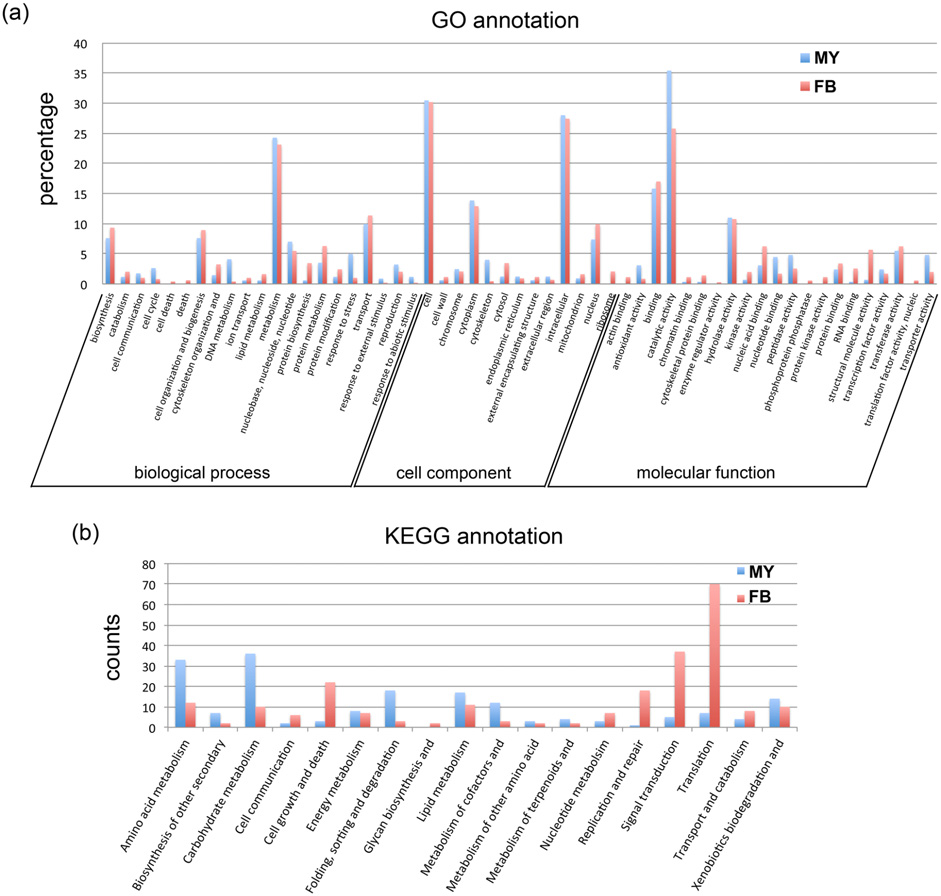
Gene ontology classification and KEGG annotation of DEGs between wild-type fruiting body and liquid cultured mycelium. MY: unigenes upregulated in liquid cultured mycelium, FB: unigenes upregulated in wild-type fruiting bodies. (a) GO annotation. 2,282 unigenes from DEGs were analyzed with blast2GO to obtain the GO terms. And the GO term were classified with CateGOrizer and separated into three major categories. (b) KEGG annotation. 2,282 unigenes from DEGs were submitted to KAAS to get the KEGG metabolic pathway classification.

To further examine the functional differences between *A*. *cinnamomea* in MY and FB, the gene ontology and KEGG annotation of the DEGs were performed with Blast2GO and KAAS (Fig 2). Wild-type fruiting bodies and liquid cultured mycelium were found to have different physiological properties, metabolic pathways and gene expression.

Several GO terms like “cell death”, “death”, “cytoskeleton organization”, “protein biosynthesis”, “protein modification” in biological processes; “cytosol”, “ribosome” in cell components; and “actin, chromosome, cytoskeletal protein and RNA binding”, “protein kinase activity” and “structural molecule activity” in molecular function were associated with genes upregulated in fruiting bodies. The KEGG annotations of DEGs were classified into different metabolic pathways. “Cell growth and death”, “nucleotide metabolism”, “replication and repair”, “signal transduction” and “translation” were upregulated in fruiting bodies. The results from the two databases were similar.

### sRNA sequencing, milRNA candidate prediction and target prediction

**Fig 3:**
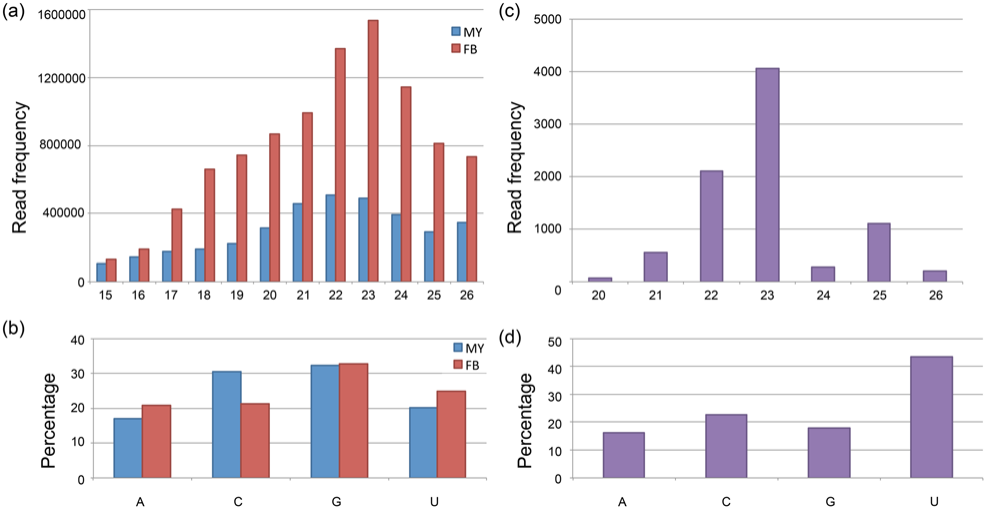
General features of the sRNAs and predicted miRNA in *A*. *cinnamomea*. (a) length distribution of total clean reads of sRNA library from MY and FB, (b) 5′ end nucleotide frequency of sRNAs from MY and FB, (c) length distribution of predicted novel milRNAs, (d) 5′ end nucleotide frequency of predicted novel milRNAs.

The general features of the sRNA library sequencing data are listed in Table 2. We obtained 14,947,280 and 23,628,123 raw reads from artificial cultured MY and wild-type FB, respectively, using the SOLiD NGS platform. After data trimming and preprocessing, we obtained 10,879,926 and 18,921,833 high quality clean reads from MY and FB, respectively. The sRNA length distribution is shown in Fig 3a and the 5′ end nucleotide frequency is shown in Fig 3b. The most frequent length was 22 and 23 nt in MY and FB, respectively. Guanine (G) was the most frequent 5′ end nucleotide.

**Table 2:** General features of small RNA sequencing from different states of *A*. *cinnamomea*.

	MY	FB	Both
Number of raw reads	14,947,280	23,628,123	-
Number of clean reads	10,879,926	18,291,833	-
Average length (nt)	28.4	26.7	-
Number of unique grouped sRNA	2,140,070	3,234,673	-
a) Number of predicted miRNA (precursor number) with conserved miRNA	58 (1)	35 (1)	128 (2)
b) Number of predicted novel milRNA (precursor number)	13 (8)	46 (16)	4 (4)

With the “Extract and Count” function in CLC Genomic Workbench, we obtained 2,140,070 and 3,234,673 final grouped sRNAs from MY and FB, respectively. The grouped sRNAs were further used for a conserved miRNA search against miRBase database version 18 using the “Annotate and Merge Counts” function in CLC Genomic Workbench. After removing the repeats, we obtained 186 and 163 annotated conserved miRNAs in the MY and FB sRNA libraries, respectively. Lastly, we took the annotated miRNAs and mapped them back to the precursors folded from RNA-seq using the RNAfold program. Only the sequences located on the stem structure were kept. We found 4 conserved predicted miRNAs (1 from MY, 1 from FB and 2 that appeared in both libraries). The sequence data and read numbers are listed in Table 3 and the mapped precursors secondary structure is showed in S2 Fig.

**Table 3:** Conserved miRNA candidates against the miRBase database.

Lib.	miR name	Sequence (5′ -3′)	Reads
F	miR-26	UUCAAGUAAUCCAGGAUAGG	2
B	miR-27d	UUCACAGUGGCUAAGUUC	6
M	miR-545	AUCAACAAACAUUUAUUGUGUG	4
B	miR-574	UGAGUGUGUGUGUGUGAGUGUGU	20

M: library from *A*. *cinnamomea* WSY-01 mycelium (MY), F: wild-type fruiting body (FB), B: in both library.

**Fig 4:**
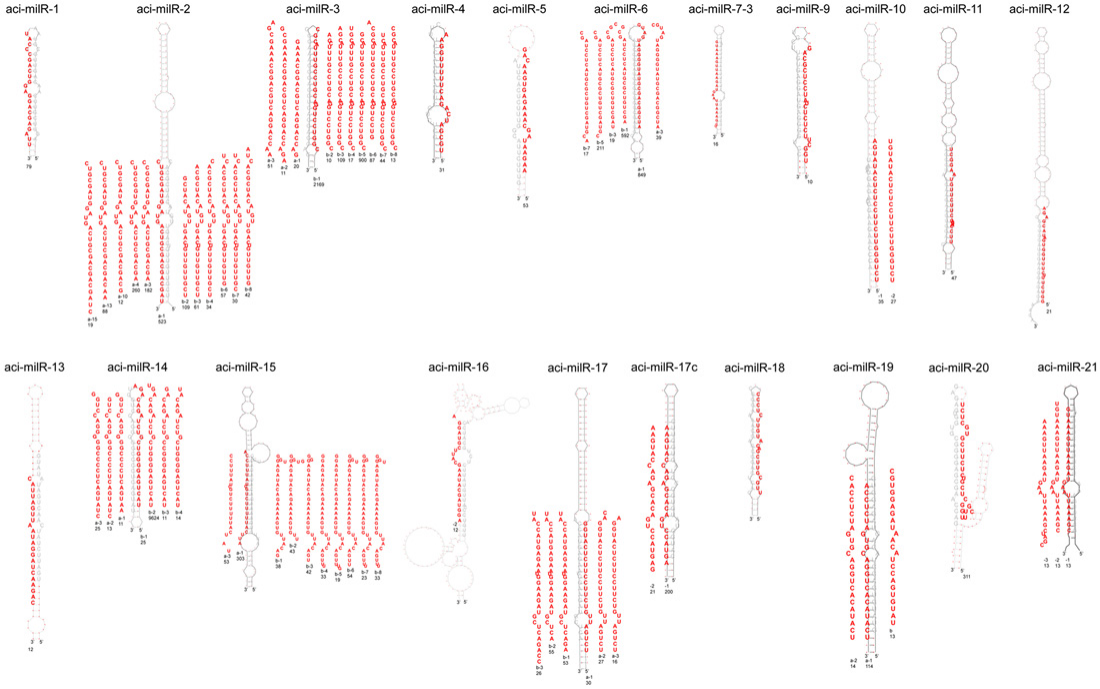
Secondary hairpin structures of milRNA from *A*. *cinnamomea* predicted by RNAfold. The milRNAs were sorted by the source of the precursors. milRNAs are marked in bold red and the reads are listed below.

**Fig 5:**
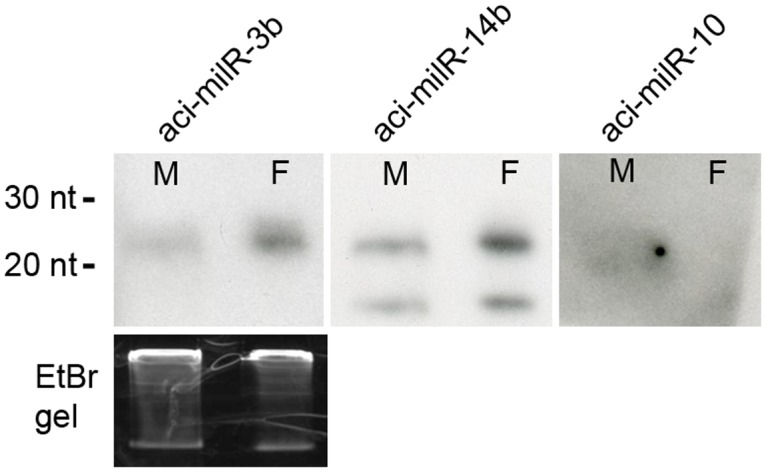
milRNAs identified in this study with Northern blot.

The final grouped sRNAs were also used to predict the milRNA candidates following the constructed pipeline. The sRNAs were re-mapped back to the folded RNA precursors with a maximum of 4 nt mismatches. After mapping, 259 and 740 possible precursor candidates in MY and FB were kept. After checking the secondary structure and the sRNA mapping condition, the results were listed in Table 2. Only 20 folded precursors (4 in MY, 11 in FB, and 5 appearing in both libraries) and 63 predicted milRNA candidates were kept. The secondary structure of the 20 folded precursors is shown in Fig 4. The milR is marked in bold red and the milR* is marked in grey. The sequence data of novel predicted milRNAs is listed in Table 4. And the milRNA length distribution is shown in Fig 3c. The most frequent length was 22–26 nt with a peak at 23 nt. The difference in length distribution may be because of the different biogenesis mechanism in fungal species compared to conventional miRNAs from animals and plants. The 5′ end nucleotide frequency of these predicted novel milRNAs shown in Fig 3d. The most frequent 5′ end nucleotide was uracil (U). Many milRNAs shared the same 5′ termini but had more nucleotides in the 3′ end. This phenomenon can also been found in *Neurospora* and other eukaryotes [7]. We also verified several milRNAs with Northern blot using isotope labeled probe and the result is showed in Fig 5. Besides aci-milR-14b have two signals, other milRNAs have the signal near the predicted nucleotide size. With the result of Northern blot, we also identified the milRNAs have different expression pattern in different development stages of *A*. *cinnamomea*.

**Table 4:** Predicted novel milRNA candidates in *A*. *cinnamomea*.

Lib.	milRNA acc.	contig_ID	milR sequence (5′-3′)	reads
F	aci-milR-1	FB_M2436	UACCCGACUGGAGGACGAGAUU	79
F	aci-milR-2a-1	FB_M2455	CUGCGAUGGAUGACUGCGACGACGAU	523
F	aci-milR-2a-3	FB_M2455	CCGCGAUGGAUGACUGCGACGA	182
F	aci-milR-2a-4	FB_M2455	CUGCGGUGGAUGACUGCGACGA	260
F	aci-milR-2a-10	FB_M2455	CUGCGAUAGAUGACUGCGACGACG	12
F	aci-milR-2a-13	FB_M2455	CUGCGAUGGAUGACUGCGACGACGC	88
F	aci-milR-2a-15	FB_M2455	CGUUGUUGUCAGUUGUACAUCGCACU	19
F	aci-milR-2b-2	FB_M2455	UCGUUGUUGUCAGUUGUACAUCG	109
F	aci-milR-2b-3	FB_M2455	UCGUUGUUGUCAGUUGUACAUCGCA	61
F	aci-milR-2b-4	FB_M2455	UCGUUGUUGUCAGUUGUACAUCGCAC	34
F	aci-milR-2b-6	FB_M2455	GUUGUUGUCAGUUGUACAUCGCACU	57
F	aci-milR-2b-7	FB_M2455	CGUUGUUGUCAGUUGUACAUCGCACU	30
F	aci-milR-2b-8	FB_M2455	GUUGUUGUCAGUUGUACAUCGCACUA	42
M	aci-milR-3a-1	FB_M4358	GAAACGGACGUCAGGACCAG	20
M	aci-milR-3a-2	FB_M4358	GCGAAACGGACGUCAGGACCAGA	11
M	aci-milR-3a-3	FB_M4358	AGCGAAACGGACGUCAGGACCAA	51
B	aci-milR-3b-1	FB_M4358	CGGUCCUGGACGUCCGUUUACGC	2169
F	aci-milR-3b-5	FB_M4358	CGGUCCUGGACGUCCGUUUACG	900
F	aci-milR-3b-6	FB_M4358	GUCCUGGACGUCCGUUUACGCA	87
F	aci-milR-3b-8	FB_M4358	CGGUCCUGGGCGUCCGUUUACGC	13
M	aci-milR-4	FB_M4900	UGCGAUCGGACUUUUUGGAA	31
F	aci-milR-5	FB_M7084	AAGAAUAGCAAAGAGUGAACAG	53
F	aci-milR-6a-1	FB_M7816	AUCGGCAGCGUAUGGGGAUGAUG	849
F	aci-milR-6a-3	FB_M7816	AUCGGCAGCGUAUGGGGAUGAUGC	39
F	aci-milR-6b-1	FB_M7816	CGCGAUCCUCAUGCGCUGUCGA	592
F	aci-milR-6b-3	FB_M7816	CGCGAUCCUCAUGCGCUGUCGAU	19
F	aci-milR-6b-5	FB_M7816	CGAUCCUCAUGCGCUGUCGAUGC	211
F	aci-milR-6b-7	FB_M7816	CGAUCCUCAUGCGCUGUCGAUGCA	17
F	aci-milR-7-3	FB_M8066	CAUCUAUGUUAAGCUAAUUGGCUUUAGUU	14
M	aci-milR-9	FB_M9694	UGGCUUCUCUCUAUCCUCGCAG	10
M	aci-milR-10-1	FB_M11279	UCUGGGUUCUUCCUCUCAUAUGA	35
M	aci-milR-10-2	FB_M11279	UCUGGGUUCUUCCUCUCAUAUGU	27
F	aci-milR-11	FB_M13321	UUCCAUCAGCUUCUUGUAAGGCU	47
F	aci-milR-12	FB_M13515	GGUCUUUCUUUGGUGAUACGAGA	21
M	aci-milR-13	FB_M15202	CAUUAGUUAAUAGGAUCAAGAC	12
F	aci-milR-14a-2	FB_M19318	GUCCAGGGGGCCCCUCAGUAUC	13
F	aci-milR-14a-3	FB_M19318	GGUCCAGGGGGCCCCUCAGUAUC	25
B	aci-milR-14b-1	FB_M19318	UACUGAGGGGUCGUCUAGACAGA	25
F	aci-milR-14b-2	FB_M19318	UACUGAGGGGUCGUCUAGACAGU	9624
M	aci-milR-15a-1	FB_M20087	CCUUUAUCGUCUUUUUACUUAUUCAAUG	25
F	aci-milR-15a-3	FB_M20087	CCUUUAUCGUCUUUUUACUUAUU	53
M	aci-milR-15b-1	FB_M20087	GACAUUGUGAAAAGACAUAGAGGGU	38
F	aci-milR-15b-2	FB_M20087	UUGUGAAAAGACAUAGAGGGUG	43
F	aci-milR-15b-3	FB_M20087	UGACAUUGUGAAAAGACAUAGAGGG	42
F	aci-milR-15b-4	FB_M20087	UUUGACAUUGUGAAAAGACAUAGAGG	22
B	aci-milR-15c-1	FB_M20087	UAUCGAUCCUUUAGUCCCUC	14
F	aci-milR-16-2	FB_M24188	AGGAUGCUUACGAAUACGAUGGA	12
M	aci-milR-17a-1	FB_M28577	UCUGAUUGUCUUCCUUCUCAUGG	30
M	aci-milR-17a-2	FB_M28577	UCUGAUUGUCUUCCUUCUCAUGGAC	27
F	aci-milR-17a-3	FB_M28577	UCUGAUUGUCUUCCUUCUCAUGGA	16
B	aci-milR-17b-1	FB_M28577	UACCAUGAGAAUGGAAGAUGCUCAGA	53
F	aci-milR-17b-2	FB_M28577	UACCAUGAGAAUGGAAGAUGCUCA	55
F	aci-milR-17b-3	FB_M28577	CAUGAGAAUGGAAGAUGCUCAGACC	26
F	aci-milR-17c-1	FB_M28577	AAGUACCAGAGCACAGUCCAUGA	200
F	aci-milR-17c-2	FB_M28577	AAGUACCAGAGCACAGUCCAUGAG	21
F	aci-milR-18	FB_M33490	UUGCGUUCGACUUGUUCUCCC	105
M	aci-milR-19a-1	FB_M33646	ACCUCUAGUGCAGGUCACAUACU	114
F	aci-milR-19a-2	FB_M33646	CACCUCUAGUGCAGGUCACAUACU	14
F	aci-milR-19b	FB_M33646	UAUGUGACCUACAAUAGAGGUGC	13
F	aci-milR-20	FB_M36986	CGUUUGGCUCGUCUCUUCGUGCUCU	311
F	aci-milR-21-1	FB_M47647	GUAAAGUUAAGAUGAAUUAAAGC	13
F	aci-milR-21-2	FB_M47647	UGUAAAGUUAAGAUGAAUUAAAGC	13
F	aci-milR-21-3	FB_M47647	AAAGUUAAGAUGAAUUAAAGCUAGA	13

M: *A*. *cinnamomea* WSY-01 mycelium (MY) library, F: wild-type fruiting body library (FB), B: in both libraries.

The predicted novel milRNAs and the related precursors were also mapped back to the genomic DNA library set up in this study. The results are listed in S2 Table. All the precursors can find the relative source on the genomic DNA sequence. Most of the milRNAs were distributed on the intergenic region, with one on the intron and one on the antisense frame of an exon. Although most of the adjacent genes remain unknown or are hypothetical proteins, several neighboring genes were predicted to be involved in gene regulation and transporters, such as P-loop containing nucleoside triphosphate hydrolase protein, WD repeat-containing protein and MFS general transporter.

The annotated conserved miRNA and novel predicted milRNA candidates were collected and used for the target prediction against the unigenes of *A*. *cinnamomea* RNA-seq. The prediction was performed with the psRNAtarget server. The results are listed in Table 5. The unigenes targeted by conserved miRNAs and predicted milRNAs from MY which had DEG with expression levels in MY lower than in FB and vice versa, were kept. Several hydrolases, proteinases, peroxidases and transferases were regulated by the predicted miRNAs and milRNAs. Most interestingly, aci-milR-9 targeted to a sterol reductase involved in the tri-terpenoid and sterol synthesis pathway, aci-milR-10 targeted a fungal pheromone receptor for recognizing the mating type, aci-milR-2b targeted to a transient receptor potential (TRP) domain containing protein that may participate in sensory of chemical or physical stimuli, and aci-milR-6b targeted to a MFS general transporter. The alignment of the miRNAs and these target genes is shown in S3 Fig.

**Table 5:** Target prediction of conserved miRNA and novel predicted milRNA candidates.

Lib.	milR_Acc.	Target_Acc.	Target_Annotation	E-value	F_RPKM	M_RPKM	Inhibition
M	aci-miR-545	Contig_255	EIW57936.1| alpha/beta-hydrolase	2E-122	63.66434	9.62542	Cleavage
M	aci-miR-545	Contig_7265	no hit		10.59487	0	Translation
M	aci-milR-4	Contig_3482	CCL99720.1| predicted protein	4E-93	45.81264	20.67553	Cleavage
M	aci-milR-4	First_Contig1064	no hit		18.17605	2.42455	Cleavage
M	aci-milR-9	First_Contig840	EIW59605.1| ERG4/ERG24 ergosterol biosynthesis protein	2E-45	143.57422	60.28609	Cleavage
M	aci-milR-9	Contig_758	EIM88703.1| Cloroperoxidase	1E-59	237.03534	34.76652	Translation
M	aci-milR-9	Contig_5374	CCM01456.1| predicted protein	0	32.89911	11.19648	Cleavage
M	aci-milR-10-1	Contig_6667	EJF59108.1| fungal pheromone STE3G-protein-coupled receptor	3E-138	9.55659	0	Cleavage
M	aci-milR-10-1	Contig_93	EIW64472.1| DUF726-domain-containing protein	0	58.37772	28.47582	Translation
M	aci-milR-10-2	Contig_6667	EJF59108.1| fungal pheromone STE3G-protein-coupled receptor	3E-138	9.55659	0	Cleavage
M	aci-milR-10-2	Contig_93	EIW64472.1| DUF726-domain-containing protein	0	58.37772	28.47582	Translation
M	aci-milR-15a-1	First_Contig654	no hit		15.24887	1.06614	Cleavage
M	aci-milR-15a-1	Contig_705	CCL98992.1| predicted protein	2E-134	28.36044	8.18889	Cleavage
M	aci-milR-17a-1	Contig_5351	EIW58650.1| hypothetical protein TRAVEDRAFT_58801	5E-128	18.40338	1.96923	Cleavage
M	aci-milR-17a-1	Contig_556	EIW61368.1| cysteine proteinase	0	76.18506	31.03941	Translation
M	aci-milR-17a-1	Contig_6059	AAC48526.1| gastric mucin	9E-6	21.82072	0.65503	Translation
M	aci-milR-17a-2	Contig_5351	EIW58650.1| hypothetical protein TRAVEDRAFT_58801	5E-128	18.40338	1.96923	Cleavage
M	aci-milR-17a-2	Contig_556	EIW61368.1| cysteine proteinase	0	76.18506	31.03941	Translation
F	miR-26	Contig_1702	hypothetical protein	0	23.28213	52.18297	Cleavage
F	miR-26	Contig_613	EIW62060.1| Pkinase-domain-containing protein	0	18.55729	56.07599	Cleavage
F	miR-26	Contig_4641	XP_001873789.1| glycosyltransferase family 31 protein	0	21.25023	52.95106	Translation
F	aci-milR-1	Contig_7086	CCM04121.1| predicted protein	5E-43	17.73866	36.88036	Cleavage
F	aci-milR-2a-12	Contig_2620	CCM04026.1| predicted protein	7E-173	69.71288	186.24744	Cleavage
F	aci-milR-2a-14	First_Contig419	EJF62899.1| MFS general substrate transporter	1E-19	18.8734	54.52335	Cleavage
F	aci-milR-2a-15	Contig_4981	EIW62923.1| TRP-domain-containing protein	0	13.42948	58.08298	Translation
F	aci-milR-2a-15	Contig_2620	CCM04026.1| predicted protein	7E-173	69.71288	186.24744	Cleavage
F	aci-milR-2a-16	Contig_2620	CCM04026.1| predicted protein	7E-173	69.71288	186.24744	Cleavage
F	aci-milR-2b-2	Contig_4981	EIW62923.1| TRP-domain-containing protein	0	13.42948	58.08298	Translation
F	aci-milR-2b-3	Contig_4981	EIW62923.1| TRP-domain-containing protein	0	13.42948	58.08298	Translation
F	aci-milR-2b-4	Contig_4981	EIW62923.1| TRP-domain-containing protein	0	13.42948	58.08298	Translation
F	aci-milR-5	Contig_9897	XP_003881743.1| Proteophosphoglycan ppg4, related	4E-8	2.5514	17.05794	Cleavage
F	aci-milR-6a-1	Contig_1134	EJF66508.1| ATPase V1 complex subunit C	0	33.39784	79.05716	Translation
F	aci-milR-6a-3	Contig_1134	EJF66508.1| ATPase V1 complex subunit C	0	33.39784	79.05716	Translation
F	aci-milR-6b-1	Contig_2607	EJF58244.1| MFS sugar transporter	0	62.0854	237.81036	Cleavage
F	aci-milR-6b-3	Contig_2607	EJF58244.1| MFS sugar transporter	0	62.0854	237.81036	Cleavage

M: conserved miRNAs and novel predicted milRNAs from mycelium (MY), F: from fruiting bodies (FB).

### Characterization and expression pattern of *DCL-1*, *DCL-2* and *QDE-2* genes

**Fig 6:**
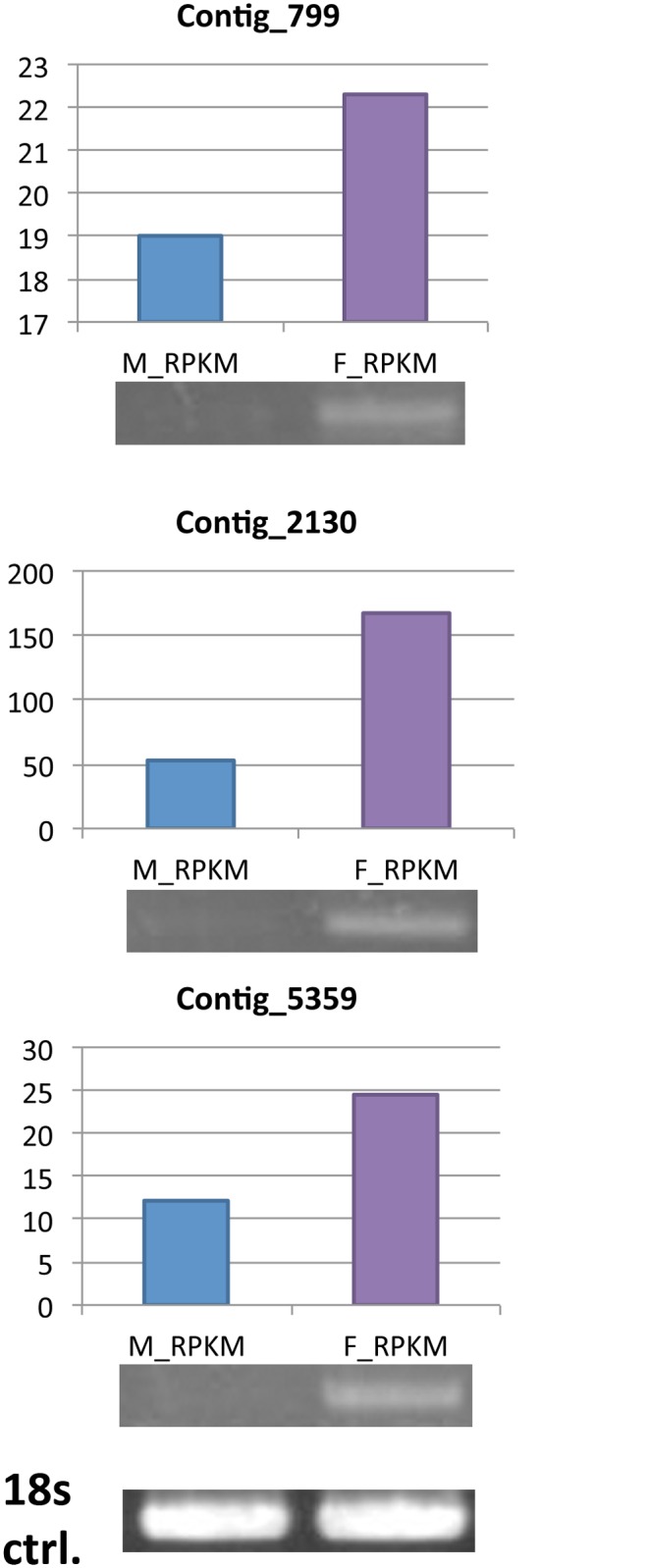
RT-PCR of *DCL-2* (Contig_799), *QDE-2* (argonaute protein, Contig_2130), *DCL-1* (Contig_5359) and 18s rRNA genes in *A*. *cinnamomea*.

The *DCL-1*, *DCL-2* and *QDE-2* gene homologs in *A*. *cinnamomea* were searched by using the BLAST function in CLC Genomic Workbench against to the unigenes from RNA-seq. Three contigs with putative function: *DCL-2* (Contig_799), *QDE-2* (argonaute protein, Contig_2130), *DCL-1* (Contig_5359) were found and the RT-PCR result is shown in Fig 6. The RPKM of these three contigs is also shown in Fig 6. Isolation of these three genes confirmed the correctness of the data mined from *A*. *cinnamomea* RNA-seq, while the higher level of expression in wild-type fruiting bodies than mycelium may also be related to the much higher expression levels and amounts of sRNAs in fruiting bodies.

## Discussion

*A*. *cinnamomea* is a host-specific, saprophyte fungus with an extremely slow rate of growth. As mentioned above, *A*. *cinnamomea* has proven pharmaceutical activity; however, many important bioactive compounds found in the wild-type fruiting bodies are reported to be rarely present in the artificial cultured mycelium. Even though several studies have focused on the improvement of the artificial culture system little progress has been made. The only host of *A*. *cinnamomea* is *C*. *kanehirai* Hayata, an endangered species in Taiwan that is currently often the target of illegal logging. To promote sustainable forest management and benefit human medical welfare, more basic knowledge about the genetics and the secondary metabolite synthesis pathways of *A*. *cinnamomea* is needed.

In recent years, over 24,000 miRNAs have been identified in animals, plants, viruses and unicellular organisms [41]. But small numbers of milRNAs have been reported in fungal species. It may be possible that miRNAs in fungal species only appear under specific developmental conditions, or the amount of miRNAs expressed is very low so traditional sequencing and characterization methods do not detect them [9]. This is the first report of milRNAs in a higher basidiomycete. High-throughput data generation using NGS techniques can improve the identification of sRNAs with low levels of expression. And the pipeline used in this study may aid in the discovery of novel milRNAs.

It is well known that miRNAs play key roles in a wide range of cellular processes through regulating post-transcriptional gene expression. In this study, we identified 4 predicted conserved miRNAs and 63 novel predicted milRNAs from two states of *A*. *cinnamomea*. Through target prediction, several important key enzymes or regulatory factors were predicted to be regulated by the miRNAs and have significant differential gene expression patterns in the two states of *A*. *cinnamomea*.

The aci-milR-9 isolated from mycelium, targeted an ERG4/ERG24 ergosterol biosynthesis protein which is an enzyme also called sterol δ 24(28)-reductase that is involved in the sterol biosynthesis pathway [42] and has higher levels of expression in fruiting bodies. The tri-terpenoid and sterol synthesis pathways share several enzymes. OSC, an important key enzyme involved in tri-terpenoid and sterol biosynthesis pathways also had higher expression in fruiting bodies. Many enzymes involved in the terpenoid backbone synthesis pathway also showed upregulated expression in fruiting bodies (data not shown). And all these results correlated to the concentrations of tri-terpenoids that are reported to be 10- to 30-fold higher in wild-type fruiting bodies compared to in submerged cultured mycelium [43].

aci-milR-10 isolated from mycelium was targeted to a fungal pheromone STE3G-proetein-coupled receptor protein. In *Saccharomyces cerevisiae*, activation of STE3 receptors targets a G protein complex and induces a signaling cascade. This signaling is important in regulating inter-cellular communication in many fungal species and plays a critical role in interaction between mating partners during the progress of sexual reproduction [44]. This targeted gene was expressed at much high levels in fruiting bodies, since sexual identification is needed for further germination of basidiospores. Such recognition is not required in in already mated haploid mycelium [45].

aci-milR-2b targeted to a TRP-domain-containing protein. Transient receptor potential (TRP) superfamily of cation channels displays more diverse activation mechanisms and selectivity than other ion channels. And the TPR containing protein plays critical roles in sensory physiology, cellular viability, and is involved in cell growth and cell wall synthesis [46,47].

aci-milR-6b targeted to a MFS sugar transporter. The major facilitator superfamily (MFS) is a group of secondary transporters with over 10,000 members which is found in all kingdoms of life. MFS transporters are involved in cell growth and homeostasis but most of their functions have not been characterized sufficiently [48,49]. The higher expression of TRP-domain proteins and MFS transporters in mycelium may be because the growing mycelium needs to locate the nutrients for absorption.

## Conclusion

In this study, NGS approaches were used to construct a reliable database to study *A*. *cinnamomea* gene expression patterns. Further, we used a previously constructed sRNA prediction pipeline to help us to discover novel milRNAs from this non-model species. Target prediction revealed several interesting genes that may be candidates for improvement in triterpenoids and secondary metabolite synthesis, as well as regulatory factors of mycelium growth and the sexual regulation. This information will aid in explaining the physiological properties of fruiting body transformation, bioactive metabolites biogenesis mechanism and enhance the industrial process of *A*. *cinnamomea*, as well as aiding the design of sustainable forest management strategies.

## Supporting Information

S1 Fig K-mer frequency distribution. The K-mer frequency distribution analysis was use to evaluate the predicted size of the genome. The predicted genome size of *A*. *cinnamomea* S28 was: 45.58 Mb.
(TIF)

S2 Fig Secondary hairpin structures of conserved miRNA from *A*. *cinnamomea* predicted by RNAfold. (TIF)

S3 Fig Alignment of the milRNAs and the related target genes. (TIF)

S1 Table Annotation of specify contigs after DEGs analysis. (DOCX)

S2 Table milRNAs distribution on *A*. *cinnamomea* S28 gDNA. (DOCX)
