# Supplementary figures and images for: MicroRNA-Like Small RNAs Prediction in the Development of Antrodia cinnamomea
Source: PLoS One. 2015 Apr 10;10(4):e0123245. doi: 10.1371/journal.pone.0123245 (PMC4393119; doi:10.1371/journal.pone.0123245)

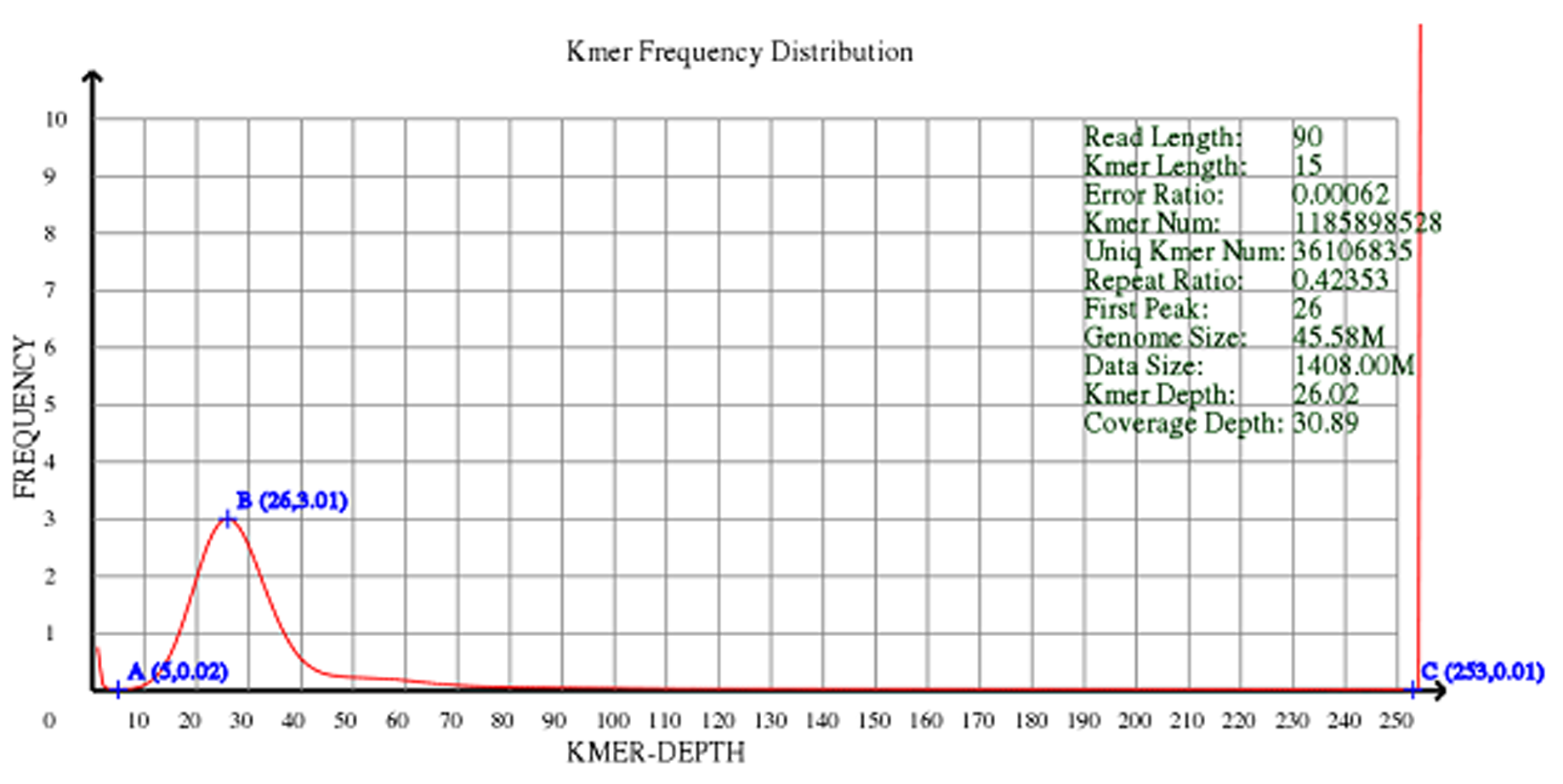

Supplement: S1 Fig — The K-mer frequency distribution analysis was use to evaluate the predicted size of the genome. The predicted genome size of A. cinnamomea S28 was: 45.58 Mb. (TIF) [file pone.0123245.s001.tif]

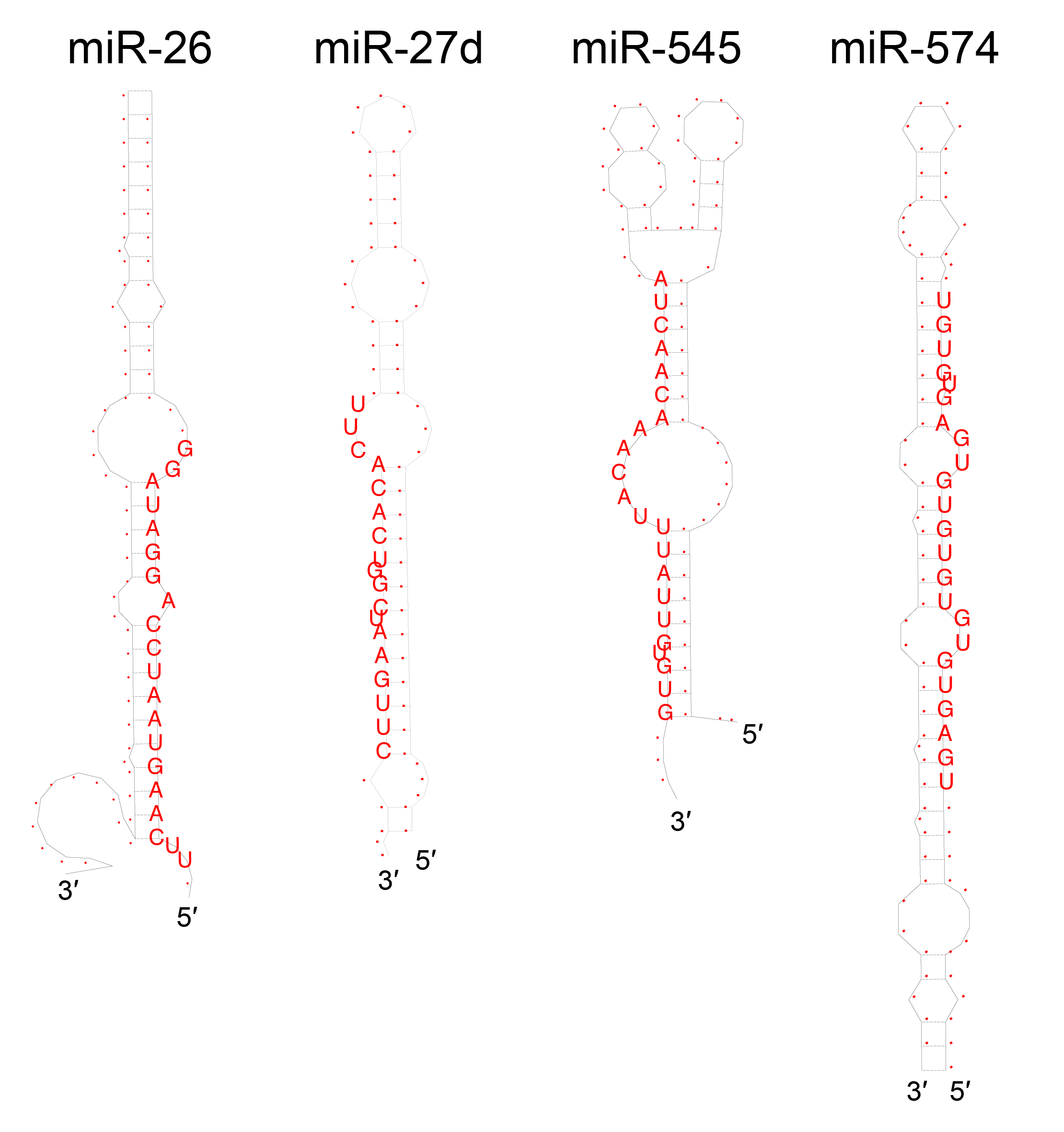

Supplement: S2 Fig — (TIF) [file pone.0123245.s002.tif]

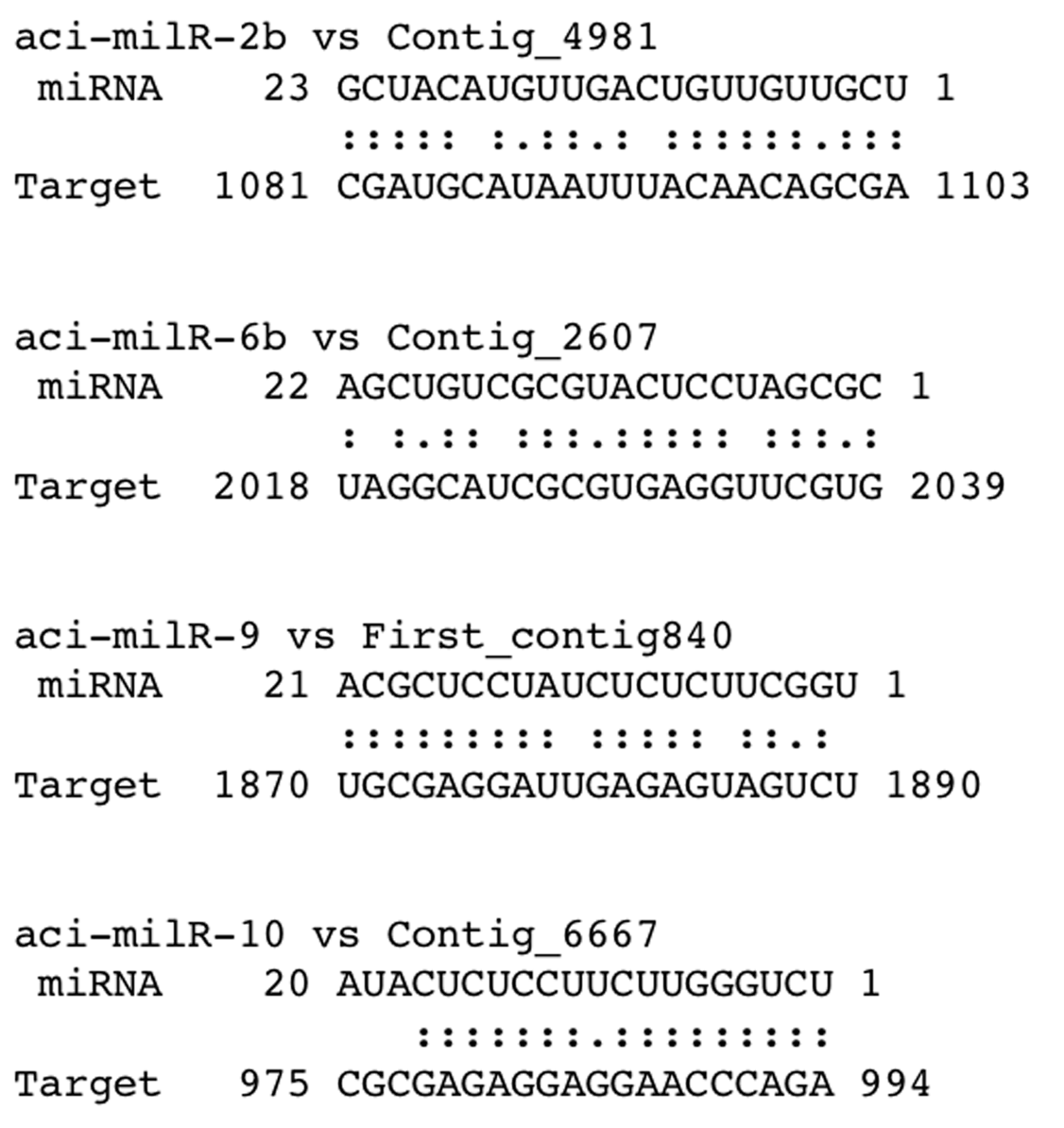

Supplement: S3 Fig — (TIF) [file pone.0123245.s003.tif]
