# Supplementary material for: MicroRNA-Like Small RNAs Prediction in the Development of Antrodia cinnamomea
Source: PLoS One. 2015 Apr 10;10(4):e0123245. doi: 10.1371/journal.pone.0123245 (PMC4393119; doi:10.1371/journal.pone.0123245)
Supplement: S1 Table — (DOCX) [file pone.0123245.s004.docx]

S1 Table. Annotation of specify contigs after DEGs analysis.

| Unigene ID | Length (bp) | Description | GI number | E-value |
| --- | --- | --- | --- | --- |
| Wild-type fruiting body expressed specify | | | | |
| Contig_459 | 2205 | homeodomain protein 1 | 382367078 | 7.0E-11 |
| Contig_3276 | 441 | alcohol dehydrogenase | 169861061 | 2.0E-17 |
| Contig_3748 | 438 | cytochrome b2, partial | 380479197 | 2.0E-14 |
| Contig_4277 | 2222 | homeodomain protein 1 | 382367078 | 2.0E-14 |
| Contig_5673 | 208 | protein kinase | 145251161 | 4.0E-21 |
| Contig_5709 | 2410 | A2 mating-type protein | 307950952 | 7.0E-37 |
| Contig_6295 | 1476 | fungal pheromone STE3G-protein-coupled receptor, partial | 395326700 | 2.0E-148 |
| Contig_6354 | 383 | ribonuclease H-like protein | 390597543 | 2.0E-27 |
| Contig_6437 | 307 | MFS general substrate transporter | 390597831 | 1.0E-12 |
| Contig_6667 | 1600 | fungal pheromone STE3G-protein-coupled receptor | 395326701 | 3.0E-138 |
| Contig_6722 | 243 | Galactokinase | 390600719 | 9.0E-15 |
| Contig_7124 | 268 | NAD-P-binding protein | 392559136 | 6.0E-29 |
| Contig_7171 | 377 | nucleic acid-binding protein, partial | 392563091 | 5.0E-10 |
| Contig_7583 | 329 | MFS general substrate transporter | 395329857 | 1.0E-64 |
| Contig_7740 | 2502 | A2 mating-type protein | 307950952 | 1.0E-33 |
| Contig_8428 | 836 | membrane-associated proteins in eicosanoid and glutathione metabolism | 395334822 | 3.0E-49 |
| Contig_8585 | 646 | predicted protein | 242208799 | 1.0E-80 |
| Contig_8613 | 624 | Rds1 protein | 392570456 | 4.0E-11 |
| Contig_9010 | 1515 | DDE-domain-containing protein | 395324988 | 6.0E-24 |
| Contig_9041 | 282 | hydroxyproline-rich glycoprotein DZ-HRGP | 6523547 | 1.0E-19 |
| Contig_9332 | 247 | thiamine pyrophosphokinase Thi80 | 170098921 | 2.0E-13 |
| Contig_10442 | 244 | protein prenylyltransferase | 392561808 | 5.0E-08 |
| Contig_10841 | 261 | predicted protein | 403418203 | 3.0E-18 |
| Contig_11596 | 364 | allantoate permease | 358370715 | 1.0E-25 |
| Contig_11935 | 203 | candidate eukaryotic translation initiation factor 4G | 242211128 | 5.0E-25 |
| Liquid cultured mycelium expressed specify | | | | |
| Contig_2980 | 540 | dipeptidyl aminopeptidase | 395325640 | 4.0E-81 |
| Contig_5322 | 232 | FAD dependent oxidoreductase | 395325630 | 2.0E-07 |
| Contig_14238 | 2544 | A2 mating-type protein | 307950952 | 3.0E-36 |
| Contig_14240 | 344 | serine-tRNA ligase | 395329294 | 2.0E-40 |
| Contig_14260 | 1145 | cytochrome b5 | 395329720 | 5.0E-73 |
| Contig_14264 | 2287 | homeodomain protein 1 | 382367078 | 8.0E-14 |
| Contig_14271 | 1453 | glycoside hydrolase family 2 protein | 390601850 | 2.0E-8 |
| Contig_14274 | 408 | hypothetical protein TRAVEDRAFT_24919 | 392558697 | 1.0E-06 |
| Contig_14281 | 2504 | A2 mating-type protein | 307950952 | 1.0E-22 |
| Contig_14288 | 1503 | fungal pheromone STE3G-protein-coupled receptor, partial | 395326700 | 5.0E-153 |
| Contig_14299 | 761 | sulfate anion transporter | 390595085 | 6.0E-134 |
| Contig_14308 | 1510 | fungal pheromone STE3G-protein-coupled receptor | 395326701 | 4.0E-133 |
| Contig_14314 | 2346 | hypothetical fungal pheromone GPCR, STE3-type | 242210647 | 7.0E-164 |
| Contig_14320 | 214 | GI19951 | 195119902 | 1.0E-19 |
| Contig_14340 | 505 | high nitrogen upregulated cytochrome P450 monooxygenase 2, partial | 393216887 | 2.0E-18 |
| Contig_14377 | 395 | NAD-P-binding protein | 392559136 | 2.0E-27 |
| Contig_14385 | 205 | DUF1769-domain-containing protein | 392561844 | 9.0E-10 |
| Contig_14386 | 690 | hypothetical protein SERLA73DRAFT_158294 | 336375469 | 4.0E-71 |
