# Supplementary material for: MicroRNA-Like Small RNAs Prediction in the Development of Antrodia cinnamomea
Source: PLoS One. 2015 Apr 10;10(4):e0123245. doi: 10.1371/journal.pone.0123245 (PMC4393119; doi:10.1371/journal.pone.0123245)
Supplement: S2 Table — (DOCX) [file pone.0123245.s005.docx]

S2 Table. milRNAs distribution on *A. cinnamomea* S28 gDNA.

| Precursor | milR_Acc. | Scaffold_ID | Position | miR 5’-3’ | gDNA 5’-3’ | Feature | Forward gene | Backward gene |
| --- | --- | --- | --- | --- | --- | --- | --- | --- |
| FB_M2436 | aci-milR-1 | scaffold_54 | 54466-54445 | UACCCGACUGGAGGACGAGAUA | TACCCGACTGGAGGACGAGATT | intergenic | predicted protein | Ras domain contain predicted protein |
| FB_M2455 | aci-milR-2a-1 | scaffold_79 | 28703-28727 | UGCGAUGGAUGACUGCGACGACGAU | TGCGATGGATGACTGCGACGACGAT | intergenic | CCA66848\|related to BZZ1-Myo3/5p-Bee1p-Vrp1p actin assembly complex component | XP_007370165\|CAF1-domain-containing protein |
| FB_M2455 | aci-milR-2b-2 | scaffold_79 | 28624-28646 | UCGUUGUUGUCAGUUGUACAUCG | TCGTTGTTGTCAGTTGTACATCG | intergenic |  |  |
| FB_M4358 | aci-milR-3a-1 | scaffold_271 | 7776-7757 | GAAACGGACGUCAGGACCAG | GAAACGGACGTCAGGACCAG | intergenic | XP_008040194\|MFS general substrate transporter | XP_008040631\|sure-like protein |
| FB_M4358 | aci-milR-3b-1 | scaffold_271 | 7802-7780 | CGGUCCUGGACGUCCGUUUACGC | CGGTCCTGGACGTCCGTTTACGC | intergenic |  |  |
| FB_M4900 | aci-milR-4 | scaffold_237 | 4955-4974 | UGCGAUCGGACUUUUUGGAA | TGCGATCGGACTTTTTGGAA | intergenic | XP_007869708\|MFS general substrate transporter | XP_007363118\|pirin domain-containing protein |
| FB_M7084 | aci-milR-5 | scaffold_119 | 31297-31276 | AAGAAUAGCAAAGAGUGAACAG | AAGAATAGCAAAGAGTGAACAG | intergenic | no hit | XP_007365704\|ribose 5-phosphate isomerase |
| FB_M7816 | aci-milR-6a-1 | scaffold_79 | 1335-1313 | AUCGGCAGCGUAUGGGGAUGAUG | ATCGGCAGCGTATGGGGATGATG | intergenic | no hit | no hit |
| FB_M7816 | aci-milR-6b-1 | scaffold_79 | 1312-1291 | CGCGAUCCUCAUGCGCUGUCGA | CGCGATCCTCATGCGCTGTCGA | intergenic |  |  |
| FB_M8066 | aci-milR-7-1 | scaffold_79 |  | ACAUCUAUGUUAAGCUAAUUGGCUUUAGUU |  | not found |  |  |
| FB_M9287 | aci-milR-8 | scaffold_267 | 16678-16706 | CGCCCAGUUCGUGCGCUGGAUAUCCCGGU | CGCCCAGTTCGTGCGCTGGATATCCCGGT | exon_antisense | hypothetical protein |  |
| FB_M9694 | aci-milR-9 | scaffold_685 | 2639-2618 | UGGCUUCUCUCUAUCCUCGCAG | TGGCTTCTCTCTATCCTCGCAG | intergenic | hypothetical protein |  |
| FB_M11279 | aci-milR-10-1 | scaffold_226 | 28155-28134 | UCUGGGUUCUUCCUCUCAUAUGA | TCTGGGTTCTTCCTCTCATATG | intergenic | XP_007369490\|dihydrodipicolinate synthetase | EPT02649\|phosphatases II |
| FB_M13321 | aci-milR-11 | scaffold_70 | 7908-7930 | UUCCAUCAGCUUCUUGUAAGGCU | TTCCATCAGCTTCTTGTAAGGCT | intergenic | XP_007843039\|zinc knuckle domain proetein | predicted protein |
| FB_M13515 | aci-milR-12 | scaffold_134 | 53608-53586 | GGUCUUUCUUUGGUGAUACGAGA | GGTCTTTCTTTGGTGATACGAGA | intron | hypothetical protein |  |
| FB_M15202 | aci-milR-13 | scaffold_247 | 30983-31004 | CAUUAGUUAAUAGGAUCAAGAC | CATTAGTTAATAGGATCAAGAC | intergenic | XP_007863123\|P-loop containing nucleoside triphosphate hydrolase protein | no hit |
| FB_M19318 | aci-milR-14a-1 | scaffold_378 | 9919-9939 | GGUCCAGGGGGCCCCUCAGUAA | GGTCCAGGGGGCCCCTCAGTA | intergenic | predicted protein | XP_007366335\|WD repeat-containing protein 8 |
| FB_M19318 | aci-milR-14b-1 | scaffold_378 | 9895-9916 | UACUGAGGGGUCGUCUAGACAGA | TACTGAGGGGTCGTCTAGACAG | intergenic |  |  |
| FB_M20087 | aci-milR-15a-1 | scaffold_40 |  | ACCUUUAUCGUCUUUUUACUUA |  | not found |  |  |
| FB_M24188 | aci-milR-16-2 | scaffold_138 | 35127-35149 | AGGAUGCUUACGAAUACGAUGGA | AGGATGCTTACGAATACGATGGA | intergenic | predicted protein | hypothetical protein with tubulin binding cofactor A |
| FB_M28577 | aci-milR-17a-1 | scaffold_211 | 30845-30823 | UCUGAUUGUCUUCCUUCUCAUGG | TCTGATTGTCTTCCTTCTCATGG | intergenic | XP_007361810\|NAD(P)-binding protein | XP_--8033649\|FAD/NAD-P binding domain containing protein |
| FB_M28577 | aci-milR-17b-1 | scaffold_211 | 30776-30751 | UACCAUGAGAAUGGAAGAUGCUCAGA | TACCATGAGAATGGAAGATGCTCAGA | intergenic |  |  |
| FB_M28577 | aci-milR-17c-1 | scaffold_211 | 30806-30828 | AAGUACCAGAGCACAGUCCAUGA | AAGTACCAGAGCACAGTCCATGA | intergenic |  |  |
| FB_M33490 | aci-milR-18 | scaffold_2 | 77118-77098 | UUGCGUUCGACUUGUUCUCCC | TTGCGTTCGACTTGTTCTCCC | intergenic | XP_008036817\| acetolactate synthase | predicted protein |
| FB_M33646 | aci-milR-19a-1 | scaffold_136 | 26029-26051 | ACCUCUAGUGCAGGUCACAUACU | ACCTCTAGTGCAGGTCACATACT | intergenic | XP_007264599\| membrane transporter | no hit |
| FB_M36986 | aci-milR-20 | scaffold_171 | 8451-8475 | CGUUUGGCUCGUCUCUUCGUGCUCU | CGTTTGGCTCGTCTCTTCGTGCTCT | intergenic | XP_002471295\| WD40 repeat protein | XP_008035879\| MFS general substrate transporter |
| FB_M47647 | aci-milR-21-1 | scaffold_142 |  | GUAAAGUUAAGAUGAAUUAAAGC |  | not found |  |  |
